# Sirtuin Type 1 Mediates the Antidepressant Effect of S-Ketamine in a Chronic Unpredictable Stress Model

**DOI:** 10.3389/fpsyt.2022.855810

**Published:** 2022-05-19

**Authors:** Lanwei Hou, Jingyu Miao, Haiwei Meng, Xiao Liu, Di Wang, Yawen Tan, Chuangang Li

**Affiliations:** ^1^Research Center for Sectional and Imaging Anatomy, Department of Anatomy and Neurobiology, School of Basic Medicine, Cheeloo College of Medicine, Shandong University, Jinan, China; ^2^Research Center for Sectional and Imaging Anatomy, Department of Anatomy and Neurobiology, School of Clinical Medicine, Cheeloo College of Medicine, Shandong University, Jinan, China; ^3^Department of Anesthesiology, The Second Hospital of Shandong University, Jinan, China

**Keywords:** depression, S-ketamine, SIRT1, BDNF, synaptic ultrastructure

## Abstract

**Background:**

Major depressive disorder (MDD) refers to a mental disease with complex pathogenesis and treatment mechanism. S-ketamine exhibited high effectiveness in treating MDD. However, the pharmacological activity of S-ketamine has not been reported yet.

**Materials and Methods:**

In this study, depression-like characteristics were induced by chronic unpredictable stress (CUS). After S-ketamine (15 mg/kg) was intraperitoneally injected, the behaviors of mice were tested by conducting open-field test, elevated plus maze test, tail suspension test, and forced swimming test. Bilateral injection of sirtuin type 1 (SIRT1) inhibitor EX-527 was injected into the medial prefrontal cortex (mPFC) to upregulate the SIRT1 expression. The expression of SIRT1 and brain-derived neurotrophic factor (BDNF) was detected by conducting Western blot and immunofluorescence assays. Meanwhile, the synaptic ultrastructure was detected by transmission electron microscopy.

**Results:**

In this study, the mice showed depression-like behavior in a series of behavioral tests. After the treatment with S-ketamine, the depression-like behavior stopped. Further, the synaptic ultrastructure in mPFC, including the decreased curvature of the post synaptic density and thinning of the postsynaptic density, improved after the S-ketamine treatment. Moreover, we found that S-ketamine had the possibility of spontaneous binding with SIRT1 at the molecular level and reversed CUS-induced SIRT1 reduction. Meanwhile, a positive relationship between SIRT1 and BDNF expression in mPFC and SIRT1 inhibitor limited the role of S-ketamine in reducing the depression-like behavior and increasing the BDNF level.

**Conclusion:**

S-ketamine upregulated the SIRT1-mediated BDNF in mPFC and reversed the synaptic structural defects caused by CUS. SIRT1 is a mediator of S-ketamine in alleviating depression-like behavior.

## Introduction

Major depressive disorder (MDD) refers to a frequently occurring and recurrent psychiatric disorder characterized by low mood, anhedonia, thoughts of worthlessness, inappropriate sense of guilt, and cognitive disturbances. More than 350 million people worldwide suffer from MDD, accounting for about 4.4% of the global population (1). Currently, the antidepressant drugs rely on modulation of monoaminergic neurotransmission, but there are limitations including mainly delayed onset of clinical improvement, inevitable side effects, and limited clinical response rates (2). Therefore, it is critical to explore new mechanisms and therapeutics for depression. S-ketamine (esketamine), a racemic mixture of the ketamine, showed stronger antidepressant effects and fewer side effects compared with ketamine (3, 4). S-ketamine is already an approved antidepressant used for treatment resistant depression in the United States, with practitioners in other countries using the compound off label. Recently, an open-label study showed that S-ketamine nasal spray and a new oral antidepressant drug had long-term safety for people suffering from depression that resisted treatment (5). Therefore, it is essential to gain more insight into the mechanism of S-ketamine to explain the pharmacological action of S-ketamine.

The level of brain-derived neurotrophic factor (BDNF), a neurotrophic protein, remarkably decreases in patients with MDD, which is considered as a risk factor for depression (6, 7). Deyama and Duman review the literature demonstrated that depression is associated with reduced levels of BDNF, contributing to neuronal atrophy in the mPFC and hippocampus, and reduced hippocampal adult neurogenesis (8). And increased expression and signaling of BDNF has been repeatedly implicated in the mechanisms of both typical and rapid-acting antidepressant drugs (9), and recent findings have started to elucidate the mechanisms through which antidepressants regulate BDNF signaling (10). Importantly, Monteggia has demonstrated a critical role for BDNF in the antidepressant action of ketamine and showed that the antidepressant-like effect of ketamine was attenuated in inducible forebrain specific BDNF knockout mice (11). This data showing that ketamine induces an antidepressant effect via increasing BDNF protein levels.

Sirtuin type 1 (SIRT1) has been proved to be involved in many pathological mechanisms of depression, including glial activation, neurogenesis, circadian rhythm, inflammatory reaction and BDNF signaling (12, 13). And a large-scale MDD population study found that the locus causing the risk of major depression was located near the SIRT1 gene (14), and the SIRT1 expression was significantly downregulated in patients with MDD (15, 16). Interestingly, Fang et al. found that the level of BDNF positively correlated with SIRT1 expression in cases of developing depression (17). This evidence suggested that SIRT1 was a possible mediator of antidepressant drugs in elevating BDNF levels. However, whether S-ketamine exhibits antidepressant effects through the SIRT1-dependent BDNF pathway remains unknown.

Taken together, we hypothesized that SIRT1 was a mediator of S-ketamine in increasing the BDNF level, and S-ketamine alleviated depression symptoms through the SIRT1-dependent BDNF pathway. Therefore, mice exhibited depression-like characteristics due to chronic unpredictable stress (CUS). The antidepressant behavior was observed by conducting behavior tests. The SIRT1 and BDNF protein levels were measured by conducting biochemical tests in the mPFC of mice administered with S-ketamine.

## Materials and Methods

### Animals

The animal center at Shandong University provided C57BL/6N male mice (*n* = 100, 6 weeks old, 20 ± 2 g). The mice were housed, five mice per cage, in a room with a 12-h light, 12-h dark cycle (lights on at 09:00 h) at constant ambient temperature (23 ± 1°C) and relative humidity (45%). Food and water were available *ad libitum*. The body weight of mice was recorded daily. After 7 days of keeping mice under these conditions, the behavior of all the mice was tested. All the studies were approved by the Ethical Committee of the Cheeloo College of Medicine.

### Drugs

S-ketamine hydrochloride (C_13_H_16_CINO, 2 ml:50 mg, No. 200228BL) was obtained from Jiangsu Hengrui Medicine Co., Ltd. (Lianyungang, Jiangsu, China). Based on previous research (18, 19), the S-ketamine at a dose of 15 mg/kg was used to relieve depression-like behavior in this study. The SIRT1 inhibitor 6-chloro-2,3,4,9-tetrahydro-1H-carbazole-1-carboxamide (EX-527, C_13_H_13_CIN_2_O, CAS: 49843-98-3) were purchased from Shanghai Biyuntian Biotechnology Co., Ltd. And based on our preliminary tests, 0.5 μg EX-527 were choose to inhibit the expression of SIRT1 in mPFC.

### Experiment 1

**FIGURE 1:**
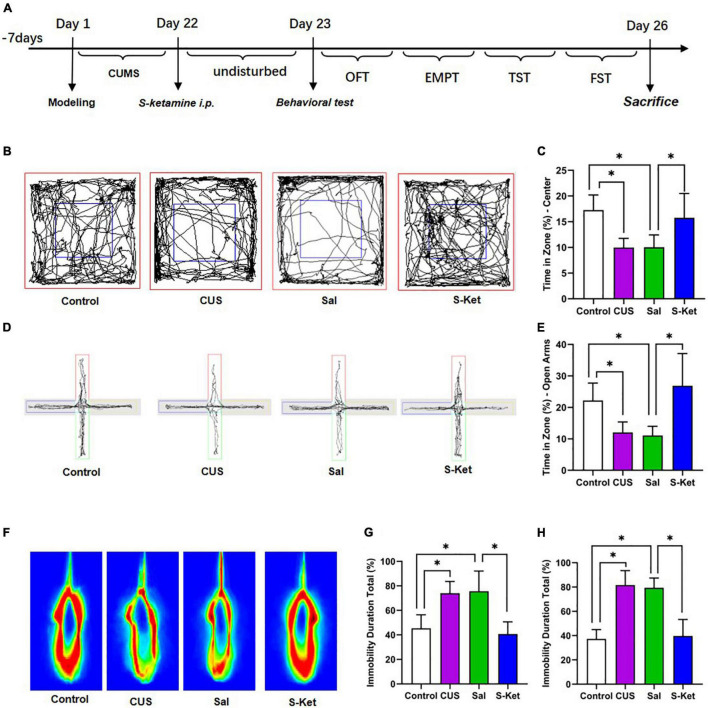
S-ketamine relieved depression-like behaviors of mice exposed to CUS. **(A)** Procedure of the experiment 1. **(B,C)** Motion trail and time of the center zone in OFT (*n* = 5–8). **(D,E)** Motion trail and time of the open arms in EPM (*n* = 5–7). **(F,G)** Percentage of immobile time in the TST (*n* = 7–8). **(H)** Percentage of immobile time in the FST (*n* = 7–9). The data are expressed as the mean ± standard error, and ^∗^*P* < 0.05.

The 40 mice used in this study were randomly divided into the following four groups to determine whether ketamine alleviated depression-like behavior by regulating SIRT1 and BDNF: control (*n* = 10): the mice received no treatment and remained in their cages for 21 days; CUS group (*n* = 10): the mice were exposed to CUS for 21 consecutive days to induce depression-like behavior as reported in previous studies; Sal group (*n* = 10): After exposure to CUS for 21 days, the mice were treated with Sal (5 mg/kg, i.p.) for a single pass; S-ket group (*n* = 10): After exposure to CUS for 21 days, the mice were treated with S-ketamine (15 mg/kg, i.p.) for a single pass. After the treatment, all the mice were subjected to behavioral and biochemical tests. The experimental procedure is shown in Figure 1A.

### Experiment 2

This study further evaluated whether SIRT1 was a mediator of S-ketamine in increasing BDNF level and alleviating depression-like behavior. The 60 mice used in this study were randomly divided into the following six groups: control (*n* = 10): The mice received no treatment and remained in their cages for 21 days; CUS group (*n* = 10): The mice were exposed to CUS for 21 consecutive days to induce depression-like behavior as reported in previous studies; Sal group (*n* = 10): After exposure to CUS for 21 days, the mice were treated with Sal (0.5 μg, injection in mPFC area) for a single pass; S-Ket group (*n* = 10): After exposure to CUS for 21 days, the mice were treated with S-ketamine (15 mg/kg, i.p.) for a single pass; EX-527 group (*n* = 10): After exposure to CUS for 21 days, the mice were treated with SIRT1 inhibitor EX-527 (0.5 μg, injection in mPFC area) for a single pass; EX-527 + S-Ket (*n* = 10): After exposure to CUS for 21 days, the mice were treated with EX-527 (0.5 μg, injection in mPFC area) and S-ketamine (15 mg/kg, i.p.) for a single pass. After the treatment, all the mice were subjected to behavioral and biochemical tests. The experimental procedure is shown in Figure 5A.

**FIGURE 2:**
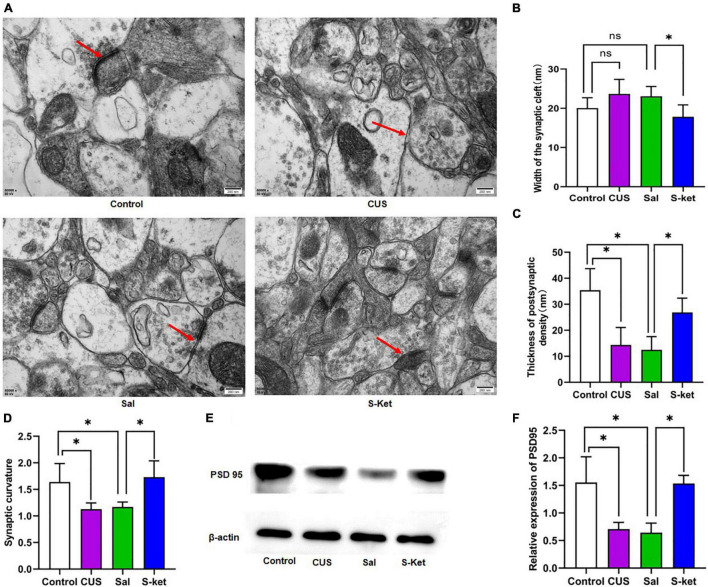
S-ketamine changed the synaptic ultrastructure in mPFC. **(A)** Synaptic ultrastructure of mPFC under ×60,000 magnification. The red arrow represents a typical structure. **(B)** Synaptic cleft width of mPFC (*n* = 3). **(C)** Thickness PSD of mPFC (*n* = 3). **(D)** Synaptic curvature of mPFC (*n* = 3). **(E)** Bands of PSD95 obtained from the Western blot test (*n* = 3). **(F)** Relative expression of PSD95 protein. The data are expressed as the mean ± standard error, and ^∗^*P* < 0.05.

**FIGURE 3:**
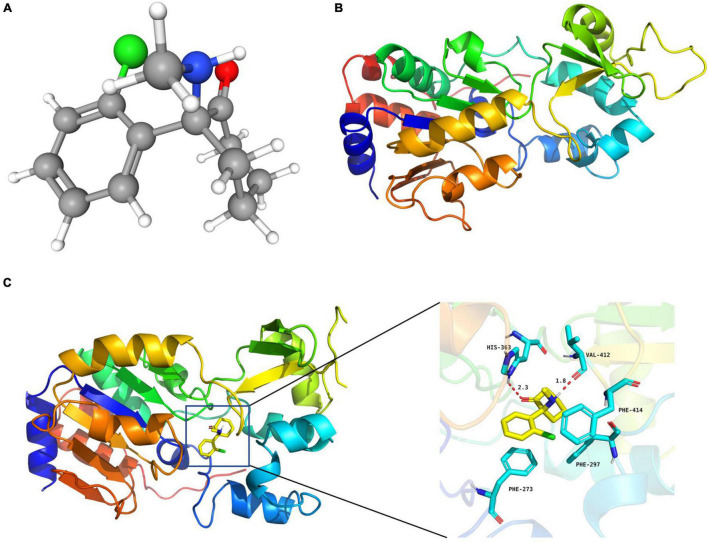
S-ketamine interacted with SIRT1 at the molecular level. **(A)** 3D structures of S-ketamine obtained from PubChem. **(B)** 3D structures of SIRT1 protein obtained from the PDB. **(C)** S-ketamine was anchored in the hydrophobic pocket of the protein.

**FIGURE 4:**
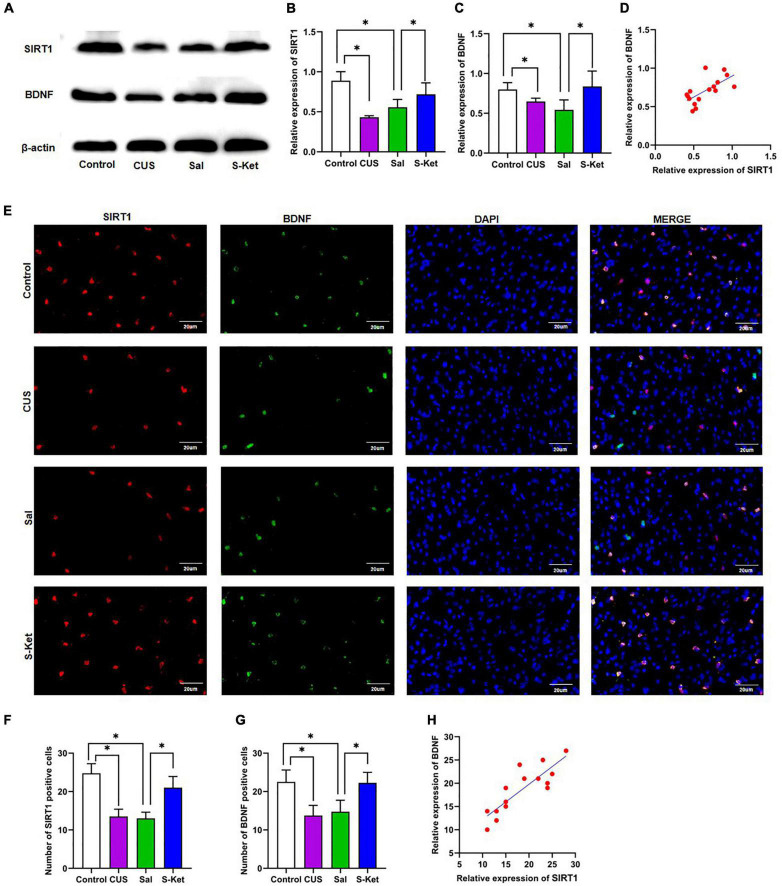
S-ketamine upregulated the SIRT1 and BDNF expression in mPFC. **(A)** Bands of SIRT1 and BDNF obtained from the Western blot test. **(B)** Relative expression of SIRT1 protein (*n* = 3). **(C)** Relative expression of SIRT1 protein (*n* = 3). **(D)** Correlation between SIRT1 protein and BDNF protein. **(E)** Images of SIRT1- and BDNF-positive cells obtained from the immunofluorescence test. **(F)** Number of SIRT1-positive cells (*n* = 3). **(G)** Number of BDNF-positive cells (*n* = 3). **(H)** Correlation between SIRT1-positive and BDNF-positive cells. The photomicrographs are typical images of SIRT1- and BDNF-positive cells (400×). The data are expressed as the mean ± standard error, and ^∗^*P* < 0.05.

**FIGURE 5:**
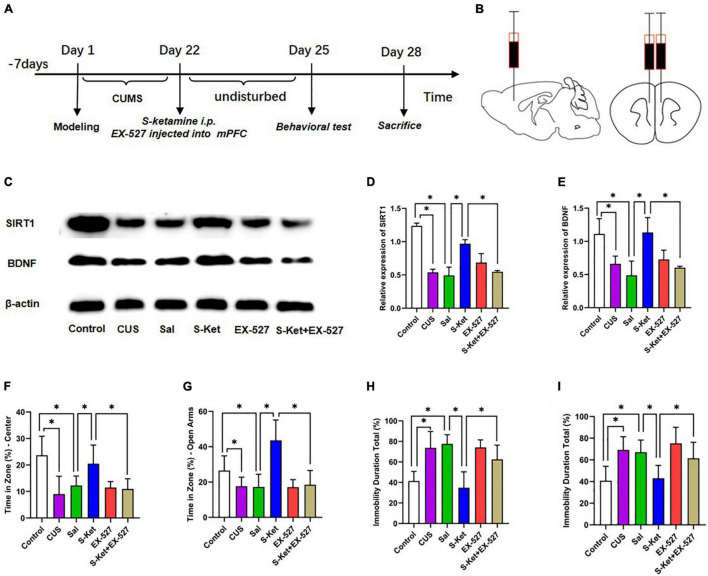
Inhibition of SIRT1 protein reversed the antidepressant effect of S-ketamine. **(A)** Procedure of Experiment 1. **(B)** Injection location of SIRT1 inhibitor EX-527. **(C)** Bands of SIRT1 and BDNF obtained from the Western blot test. **(D)** Relative expression of SIRT1 protein (*n* = 6). **(E)** Relative expression of SIRT1 protein (*n* = 6). **(F)** Motion trail and time of the center zone in OFT (*n* = 6–8). **(G)** Motion trail and time of the open arms in EPM (*n* = 5–9). **(H)** Percentage of immobile time in the TST (*n* = 8–9). **(I)** Percentage of immobile time in the FST (*n* = 7–9). The data are expressed as the mean ± standard error, and ^∗^*P* < 0.05.

### Chronic Unpredictable Stress

The depression-like behavior in mice was induced by CUS. The CUS procedure employed was a modification of published reports (20). Briefly, the mice were exposed to the following stressors: (1) water deprivation for 24 h, (2) food deprivation for 24 h, (3) cold swim at 4°C for 30 min, (4) 6-h cage tilt (45°), (5) overnight illumination, (6) restraint stress for 2 h (For restraint stress, mice were placed in 50 ml centrifuge tube with opening in one corner allowing free respiration but restricting any movement), (7) stroboscopic stimulus (150 flashes/min, 200 Lumen) for 24 h, and (8) a soiled cage environment [a soiled cage environment (200 mL water in 100 g sawdust bedding) for 24 h]. The mentioned stressors were randomly executed once daily for 21 days.

### Prefrontal Cortex Stereotaxic Surgery

Sirtuin type 1 inhibitor EX-527 was injected bilaterally into the medial prefrontal cortex (mPFC) to inhibit the expression of SIRT1 protein. Briefly, the mice were anesthetized with isoflurane and placed in a stereotaxic frame. Next, 0.5 μg EX-527 was injected bilaterally into the mPFC [coordinates: anterior–posterior (AP) = 1.8 mm, medial–lateral (ML) = ±0.4 mm, and dorsal–ventral (DV) = −2.6 mm from the bregma] (21) of mice at a rate of 0.2 μL/min using a 5-μL Hamilton syringe connected to a 30-gauge needle. Subsequently, the mice were returned to their cages under warm maintenance.

### Behavior Test

#### Open-Field Test

The open-field test (OFT) was used for assessing anxiety-like behavior in a CUS model in previous study (22). The open field comprised of an enclosed square arena made of dark opaque Plexiglas (60 cm × 60 cm) surrounded by walls (30 cm high). Each mouse was gently placed into an identical corner of the arena facing an identical direction and allowed to explore the arena for 10 min freely. The time taken by the mice in the center square and the track movement were recorded using a video camera (SMART 3.0; Panlab S.L., Barcelona, Spain). The criteria for entering the center square were defined as 50% of the body being positioned within this square.

#### Elevated Plus Maze Test

The elevated plus maze test (EPM) was used to assess anxiety-like behavior as described previously (23). After the OFT, the mice were subjected to the EPM. The EPM apparatus had two closed arms (50 cm × 10 cm with 40-cm walls), two open arms (50 cm × 10 cm without walls), and a center platform (10 cm × 10 cm). The maze was placed at the height of 50 cm above the floor. Each mouse was gently placed in the central platform, facing the closed arms, and allowed to explore the arena for 6 min freely. The time taken by the mouse in open arms and the track movement were recorded using a video camera (SMART 3.0; Panlab S. L., Barcelona, Spain). The criterion for entering the open arm was defined as 50% of the body being positioned within this arm.

#### Tail Suspension Test

The tail suspension test (TST) can reliably assess depression-like behavior. In this study, the mouse was suspended upside down by tails above the floor (60 cm) using adhesive tape placed at the height of 1 cm from the tip of the tail. The time of immobility and the motion heat map were recorded for 6 min using a video camera (SMART 3.0; Panlab S. L., Barcelona, Spain). The criterion for immobility was defined as the body being completely motionless and hanging passively.

#### Forced Swimming Test

The forced swimming test (FST) was conducted to determine depression-like behavior in mice. In this study, the mice were placed in a clear glass cylinder (25 cm high and 10 cm in diameter) filled with water of 25 cm height (24 ± 1°C). The time of immobility and the motion heat map were recorded for 6 min using a video camera (SMART 3.0; Panlab S. L., Barcelona, Spain). The criteria for immobility were defined that the animal is moving at a speed less than 40 cm/s.

### Western Blot Test

The mice were quickly decapitated, and the prefrontal lobe was separated. Subsequently, the brain tissue was homogenized using lysis buffer (RIPA, P0013K, Beyotime, Shanghai, China) and centrifuged at 12,000 rpm at 4°C for 15 min. The supernatants were reserved, and the protein samples were analyzed using sodium dodecyl sulfate–polyacrylamide gel electrophoresis. The proteins were then transferred to a polyvinylidene fluoride (PVDF, AR0136-02, Boster Biological Technology, Wuhan, China) membrane using a Trans-Blot wet transfer system (164-5050, Bio-Rad, Hercules, CA, United States). After incubating with an antigen-blocking solution for 2 h, a PVDF membrane was sequentially incubated with BDNF (AF1423, 1:1000, Beyotime, Shanghai, China), SIRT1 (AF1267, 1:1000, Beyotime, Shanghai, China), PSD95 (AF1096, 1:1000, Beyotime, Shanghai, China), and β-actin (AF5001, 1:1000, Beyotime, Shanghai, China) antibody for overnight. After washing with phosphate buffer saline (PBS) for three times, the PVDF membrane was incubated with HRP-labeled Goat Anti-Mouse IgG (H + L) (A0216, 15000, Beyotime, Shanghai, China) and HRP-labeled Goat Anti-Rabbit IgG (H + L) (A0208, 1:5000, Beyotime, Shanghai, China) for 2 h. An image was obtained after ECL (P0018S, Beyotime, Shanghai, China) development by the Amersham Imager 680 (General Electric Company, Boston, MA, United States). And the band intensities were normalized to β-actin.

### Immunofluorescence Test

After perfusion with PBS and 4% paraformaldehyde (BL539A, Biosharp, Guangzhou, China), the mouse brain was fixed with paraformaldehyde for 48 h. Subsequently, coronal cryotome sections (20 μm) were cut through the mPFC using a cryostat and washed with PBS three times. After incubating with 0.5% Triton X-100 (P0096, Beyotime, Shanghai, China) for 30 min and an antigen-blocking solution at ambient temperature for 1 h, the brain sections were sequentially incubated with BDNF (AF1423, 1:100, Beyotime, Shanghai, China) and SIRT1 (60303-1-Ig, 1:100, Proteintech Group, Inc., Wuhan, China) antibody for overnight. Next, after washing with PBS for three times, the brain sections were incubated with Alexa Fluor 488-labeled Goat Anti- Rabbit IgG (H + L) (A0428, 1:100, Beyotime, Shanghai, China) and Alexa Fluor 555-labeled Donkey Anti-Mouse IgG (H + L) (A0453, 1:100, Beyotime, Shanghai, China). The nucleus was counterstained with DAPI (AR1176, Boster Biological Technology, Wuhan, China). Ten randomly selected non-overlapping fields of x400 magnification were collected from three mPFC tissue per group. An image was obtained using a ZEISS microscope (Axioscope 5, ZEISS, Germany). And the numbers of SIRT1–BDNF–co-labeled cell were automatically measured using ZEISS Imaging Elements Software (ZEISS, Germany).

### Transmission Electron Microscopy

The mice were perfused with 30 mL of 0.1 mol/L PBS, followed by perfusion with 50 mL of fixative of an electron microscope specimen (4% paraformaldehyde + 0.1M PBS + 0.5% glutaraldehyde). Next, the brain tissue was decapitated at a low temperature, and the mPFC was bluntly dissected. The mPFC tissue (about 1 mm × 1 mm × 1 mm) was separated and fixed with 4% glutaraldehyde for 2 h and 1% osmium tetroxide for 2 h. The tissue was then embedded with epoxy resin. The hypothalamus sample was cut into slices, about 50 nm thick, and soaked in uranium dioxane acetate for 45 min for electron staining. Neuronal ultrastructure images were collected using an H-7650 transmission electron microscope. Ten synapses form three PFC tissue per group were selected for ultrastructure analysis accordance with the Guldner and Jones’ methods (24, 25).

### Molecular Docking

Molecular docking is widely used to predict ligand–target interactions at a molecular level (26). The initial three-dimensional (3D) structures of SIRT1 (code: 4I5I) were taken from Protein Data Bank (PDB)^1^. The structures of S-ketamine (Compound CID: 182137) were obtained from PubChem^2^. PyMOL (V 2.4.0) was used to dehydrate the protein, delete the original ligand, and hydrogenate and calculate the charge. The substrate ketamine was docked into the active site of protein using the AutoDock Vina tool (V1.1.2) in Chimera. The docked poses with the highest docking scores were used for further analysis. The docking result was visualized using PyMOL software.

### Statistical Analyses

All the data reported in this study were obtained by an independent investigator blinded to the experimental conditions and analyzed using SPSS (Version 22.0, SPSS, Inc., Chicago, IL, United States). The outlier (a measured value that is more than two standard deviations from the mean) would be deleted by SPSS. A one-way ANOVA followed by Dunnett’s test were used where all groups are compared to control. When comparing the Sal group with the S-ket group and S-ket group with the EX-527 + S-Ket group, the *t*-test was used. *P* < 0.05 indicated significant statistical differences. All the data were expressed as mean ± standard error using GraphPad Prism 5.0.

## Results

### S-Ketamine Relieved Depression-Like Behavior of Mice Exposed to Chronic Unpredictable Stress

In this study, after exposure to CUS for 21 days, the mice spent significantly less time in the central area of the OFT compared with the control [ANOVA: *df*_(*betweengroups*)_ = 3, *df*_(*withingroups*)_ = 22, *F* = 10.429, *P* = 0.001; *post hoc* test: *P* = 0.001; as shown in Figures 1B,C]. The use of S-ketamine increased the time in the central area compared with that in the Sal group (*t*-test: *t* = 2.918, *P* = 0.014; as shown in Figures 1B,C). Meanwhile, the time taken in open arms was significantly reduced after exposure to CUS [ANOVA: *df*_(*betweengroups*)_ = 3, *df*_(*withingroups*)_ = 20, *F* = 10.094, *P* < 0.001; *post hoc* test: *P* = 0.031; as shown in Figures 1D,E], but it was reversed by administering S-ketamine (*t*-test: *t* = 3.644, *P* = 0.005; as shown in Figures 1D,E). The immobility time in the TST experiment in the CUS group [ANOVA: *df*_(*betweengroups*)_ = 3, *df*_(*withingroups*)_ = 25, *F* = 17.325, *P* < 0.001; *post hoc* test: *P* = 0.001; as shown in Figures 1F,G] and Sal group [ANOVA: *df*_(*betweengroups*)_ = 3, *df*_(*withingroups*)_ = 26, *F* = 36.976, *P* < 0.001; *post hoc* test: *P* < 0.001; as shown in Figures 1F,G] was significantly prolonged. In contrast, the immobility time of mice treated with S-ketamine was significantly shortened (*t*-test: *t* = 5.039, *P* < 0.001; as shown in Figures 1F,G). Meanwhile, after administering S-ketamine, the mice exhibited similar behavior in the FST experiment (*t*-test: *t* = 6,832, *P* < 0.001; as shown in Figure 1H).

### S-Ketamine Changed the Synaptic Ultrastructure in Medial Prefrontal Cortex

Sirtuin type 1 mediates the synaptic ultrastructure in chronic stress-elicited depression-like phenotype (27). In this study, we found that the curvature of synaptic interface increased in the CUS group compared with the control group [ANOVA: *df*_(*betweengroups*)_ = 3, *df*_(*withingroups*)_ = 16, *F* = 7.820, *P* = 0.002; *post hoc* test: *P* < 0.015; as shown in Figures 2A,D]. This mentioned deficit was avoided using S-ketamine (*t*-test: *t* = 3.512, *P* = 0.013). Meanwhile, the PSD was downregulated in the CUS group [ANOVA: *df*_(*betweengroups*)_ = 3, *df*_(*withingroups*)_ = 17, *F* = 14.188, *P* < 0.001; *post hoc* test: *P* < 0.001; as shown in Figures 2A,C], but upregulated in the Ket group (*t*-test: *t* = 4.459, *P* = 0.011; as shown in Figures 2A,C). Moreover, the expression of PSD95 was lower in the CUS group than in the control group [ANOVA: *df*_(*betweengroups*)_ = 3, *df*_(*withingroups*)_ = 8, *F* = 10.729, *P* = 0.004; *post hoc* test: *P* = 0.027; as shown in Figures 2E,F]. S-ketamine significantly increased the PSD95 level (*t*-test: *t* = 6.807, *P* = 0.002; as shown in Figures 2E,F). However, there was no significant change in the width of the synaptic cleft in mice exposed to CUS [ANOVA: *df*_(*betweengroups*)_ = 3, *df*_(*withingroups*)_ = 8, *F* = 66.732, *P* = 0.001; *post hoc* test: *P* = 0.096; as shown in Figures 2A,B]. But after S-ketamine treatment, the synaptic cleft was significantly reduced in S-Ket group when compared with the Sal group (*t*-test: *t* = 3.986, *P* = 0.001; as shown in Figures 2A,B).

### S-Ketamine Interacted With Sirtuin Type 1 at the Molecular Level

The 3D structures of S-ketamine and SIRT1 protein are shown in Figures 3A,B. After docking, the docking binding energy between S-ketamine and the protein SIRT1 was −8.2 kcal/mol (when the binding energy was less than 0, small ligand molecules could spontaneously bind to the receptor protein). As shown in Figure 3C, S-ketamine was anchored in the hydrophobic pocket of the protein, and π-π interaction was formed between the benzene ring of Phe273 and the conjugated structure of the substrate. A stable hydrogen bond was formed between S-ketamine and His363 as well as the Val412 of SIRT1. Meanwhile, the conjugated structures of Phe297 and Phe414 formed p-π conjugation with the chlorine atom of S-ketamine.

### S-Ketamine Upregulated the Expression Level of Sirtuin Type 1 and Brain-Derived Neurotrophic Factor in Medial Prefrontal Cortex

Western blot and immunofluorescence tests were performed to explore the potential mechanism of S-ketamine production of antidepressants. As shown in Figures 4A–C, the expression levels of SIRT1 [ANOVA: *df*_(*betweengroups*)_ = 3, *df*_(*withingroups*)_ = 12, *F* = 14.709, *P* = 0.008; *post hoc* test: *P* < 0.001; as shown in Figure 4B] and BDNF [ANOVA: *df*_(*betweengroups*)_ = 3, *df*_(*withingroups*)_ = 12, *F* = 9.895, *P* = 0.001; *post hoc* test: *P* = 0.047; as shown in Figures 4D,E] were downregulated in the CUS group, and the use of S-ketamine increased the SIRT1 level (*t*-test: *t* = 3.026, *P* = 0.023; as shown in Figure 4B) and BDNF (*t*-test: *t* = 2.496, *P* = 0.047; as shown in Figure 4C) protein. Meanwhile, the expression of SIRT1 significantly positively correlated with the BDNF level (*R*^2^ = 0.4356, *P* = 0.0053; as shown in Figure 4D). Additionally, the expression of SIRT1 was co-located with that of BDNF in the immunofluorescence test (as shown in Figure 4E). S-ketamine also reversed the decrease in the number of SIRT1 (*t*-test: *t* = 4.753, *P* = 0.003; as shown in Figure 4F) and BDNF (*t*-test: *t* = 3.693, *P* = 0.010; as shown in Figure 4G) positive cells in the MPFC after exposure to CUS. Moreover, a positive relationship was observed between SIRT1- and BDNF-positive cells (*R*^2^ = 0.7311, *P* < 0.001, as shown in Figure 4H).

### Inhibition of Sirtuin Type 1 Protein Reversed the Antidepressant Effect of S-Ketamine

Sirtuin type 1 inhibitor EX-527 was used to downregulate the SIRT1 expression to further evaluate whether SIRT1 was a mediator of S-ketamine in alleviating depression-like behavior. As shown in Figure 5, after treatment with EX-527, the SIRT1 level decreased [ANOVA: *df*_(*betweengroups*)_ = 5, *df*_(*withingroups*)_ = 18, *F* = 50.823, *P* < 0.001; *post hoc* test: *P* < 0.001; as shown in Figure 5D], and the effect of S-ketamine on increasing the SIRT1 level was also inhibited (*t*-test: *t* = 13.485, *P* < 0.001; as shown in Figure 5D). Interestingly, the expression of BDNF showed an identical trend with the change in SIRT1 expression (*t*-test: *t* = 4756, *P* = 0.003; as shown in Figure 5E). And result shown in the Supplementary Figure 1, when treated with S-ketamine, it can increase the weight of mice with CUS. And after using of the EX-527, the weight gain of S-ketamine was reversed. Meanwhile, after EX-527 injection, the antianxiety-like characteristics of S-ketamine reduced in both in OFT (*t*-test: *t* = 3.512, *P* = 0.005; as shown in Figure 5F) and EPM (*t*-test: *t* = 4.489, *P* = 0.001; as shown in Figure 5G). In addition, bilateral injection of EX-527 into the mPFC reversed the effect of S-ketamine in relieving the depression-like characteristics of mice in both TST (*t*-test: *t* = 3.836, *P* = 0.002; as shown in Figure 5H) and FST (*t*-test: *t* = 3.181, *P* = 0.006; as shown in Figure 5I).

## Discussion

Depression is a mental disease with a high incidence and low response rate to drug treatment (1). Numerous studies have shown that S-ketamine exhibits a stronger antidepressant effect (3–5). In this study, we found that S-ketamine upregulated the SIRT1 and BDNF expression in mPFC. Meanwhile, the use of S-ketamine reversed the synaptic structural defects caused by CUS, and the inhibition of SIRT1 protein reversed the antidepressant effect of S-ketamine. The results indicated that SIRT1 was a mediator of S-ketamine in alleviating depression-like behavior. This mechanism of S-ketamine in alleviating depression has not been reported.

CUS protocol is a robust animal model with similar effects of environmental factors on human depression, strongly leading to reduced spontaneous exploratory activity and depression-like behavior (the main symptom of depression characteristics) in mice (28, 29). In this study, the mice showed reduced spontaneous activity and anxiety-like characteristics in OFT and EPM. Meanwhile, the immobility time of mice exposed to CUS increased, indicating that mice exhibited depression-like behavior. The results indicated the successful replication of a mouse model of depression based on the CUS protocol. After treatment with S-ketamine, the depression-like behavior could be avoided, indicating that S-ketamine also relieved depression-like symptoms in mice. The results were consistent with those obtained by Elmira (30).

Sirtuin type 1, identified as one of the two genome-wide significant loci that contribute to depression (14), has now become a critical therapeutic target (12). The existing studies showed that SIRT1 expression in the serum obtained from patients with depression decreased compared with healthy people (16, 31). As proven by animal studies, chronic stress leads to reduced SIRT1 activity and elevated risk in terms of depression-like characteristics (21, 32). Interestingly, the results of the molecular docking shown s-ketamine has the possibility of spontaneous binding with SIRT1 and they can form stable complexes, which implied SIRT1 may be the target of ketamine. Afterward, we further explored whether ketamine affected the expression level of SIRT1, and we found that the SIRT1 level decreased within the mPFC of mice exposed to CUS, and this defect was reversed using S-ketamine. Meanwhile, SIRT1 inhibitor reversed the effect of S-ketamine in relieving depression-like behavior. The results showed that SIRT1 was a mediator of S-ketamine in alleviating depression-like behavior. However, we did not determine which cells were SIRT1 positive in this study. Therefore, we will further analyze whether SIRT1 positive cells are neurons, astrocytes or microglial cell, and clarify whether the differential expression of SIRT1 is cell-specific.

SIRT1 participates in depression through various mechanisms, covering inflammation (33), BDNF signal (34), and neuronal excitability (21). Especially, Liu et al. found that SIRT1 regulated depression-like characteristics under the induction from chronic stress via BDNF in mice (32). The administration of ketamine also resulted in antidepressant effects by increasing the expression of hippocampal BDNF (35). In this study, a positive relationship was observed between SIRT1 and BDNF expression in mPFC, and SIRT1 inhibitor decreased the BDNF level elevated using S-ketamine. The result indicated that SIRT1 regulated depression-like behavior by regulating the BDNF expression, and SIRT1 controlled the S-ketamine-evoked release of BDNF in the mPFC of mice, providing a new explanatory mechanism for S-ketamine antidepressant. Several studies showed that SIRT1 reduced the CREB activity and the binding of CREB to the mBDNF promoter, thereby inducing the reduction of BDNF at the mRNA and protein levels (36, 37). Moreover, El Hayek et al. reported that SIRT1 also mediated BDNF expression by increasing the level of transcriptional coactivator PGC1a and the secreted molecule FNDC5 (34). Therefore, multiple mechanisms of action might exist between SIRT1 and BDNF, which will be further explored and elucidated in our subsequent experiments. Notably, existing literature suggests that the hippocampus is also a key region in which ketamine exerts its antidepressant effects and S-ketamine has pro-neuroplastic effects on hippocampal structures (38, 39). Meanwhile, SIRT1 activation in hippocampus blocked both the development of depression-related phenotypes and aberrant dendritic structures elicited by chronic stress exposure (32, 40). In this study, we found that the expression level of SIRT1 in hippocampus was also decreased in CUS exposed mice and this defect has been reversed by S-ketamine treatment (see Supplementary Figure 2). So, in future experiments, we will continue to explore the effects of S-ketamine on many brain regions, including the hippocampus.

Synaptic structures can respond adaptively to changing environments, and the structural remodeling of neurons occurs after stress, participating within cognitive deficits and depression (27, 41). The mice exposed to CUS exhibited reduced synaptic transmission, including PSD thinning and synaptic cleft widening (27). With the increase in the width of the synaptic cleft, the neurotransmitter delivery from the presynaptic membrane to the postsynaptic membrane could be hindered (42). PSD stopped the communication among neurons, showing a relationship between the response of the postsynaptic membrane and the signal (43). We found an increase in PSD thinning after exposure to CUS, but no significant changes in synaptic cleft. This situation was improved after treatment with ketamine, indicating that S-ketamine could remodel the synaptic ultrastructure correlated with the depression-like characteristics. SIRT1 and BDNF participated in the mediation of synaptic plasticity and synaptic structure remodeling (27, 44). Therefore, based on the results obtained in this study, we hypothesized that S-ketamine mediated synaptic structure through SIRT1 and BDNF in depression.

## Conclusion

This study evaluated a new mechanism by which ketamine ameliorated depression-like behavior. We found that S-ketamine upregulated the SIRT1 and BDNF expression in mPFC. Meanwhile, the use of S-ketamine reversed synaptic structural defects caused by CUS. Moreover, SIRT1 was a mediator of S-ketamine in alleviating depression-like behavior. This study provided more insights into the action mechanism of ketamine and might help identify new drug targets.

## Data Availability Statement

The raw data supporting the conclusions of this article will be made available by the authors, without undue reservation.

## Ethics Statement

The animal study was reviewed and approved by the Ethical Committee of the Cheeloo College of Medicine.

## Author Contributions

LH, JM, and HM developed the concept of the manuscript. LH wrote the first draft of the manuscript. XL, DW, and CL were responsible for data and manuscript review. All authors read and approved the final manuscript.

## Conflict of Interest

The authors declare that the research was conducted in the absence of any commercial or financial relationships that could be construed as a potential conflict of interest.

## Publisher’s Note

All claims expressed in this article are solely those of the authors and do not necessarily represent those of their affiliated organizations, or those of the publisher, the editors and the reviewers. Any product that may be evaluated in this article, or claim that may be made by its manufacturer, is not guaranteed or endorsed by the publisher.

## Footnotes

1
http://www.rcsb.org

2
https://pubchem.ncbi.nlm.nih.gov/
