# Supplementary material for: Sirtuin Type 1 Mediates the Antidepressant Effect of S-Ketamine in a Chronic Unpredictable Stress Model
Source: Front Psychiatry. 2022 May 19;13:855810. doi: 10.3389/fpsyt.2022.855810 (PMC9160425; doi:10.3389/fpsyt.2022.855810)
Supplement: Supplementary file 1 [file Data_Sheet_1.docx]

**Supplementary Figure 1**

Result shown the after-ketamine treatment, the body weight is increased. And after using of the EX-527, the weight gain of s-ketamine was reversed. This result shown that inhibition of SIRT1 protein reversed the weight gain of s-ketamine.


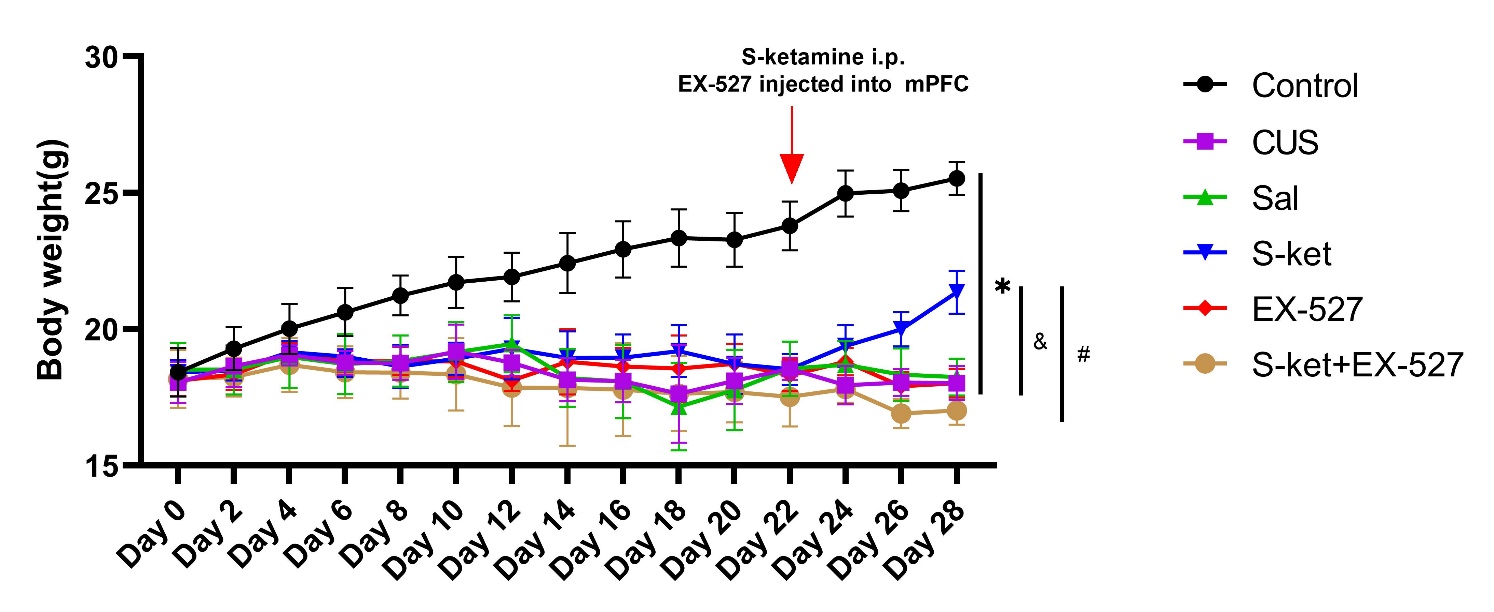


Supplementary Figure 1. Body weight changes in each group. The data are expressed as the mean ± standard error. * *P* < 0.001: control group vs CUS group; & *P* < 0.01: Sal group vs S-ket group; # *P* < 0.001: S-ket group vs S-ket +EX-527 group;

**Supplementary Figure 2**

As shown in Supplementary Figure 2, the expression levels of SIRT1 (ANOVA: *df* _(between groups)_ = 3, *df* _(within groups)_ = 8, *F* = 33.063, *P* < 0.001; *post hoc* test: *P* < 0.004; as shown in Supplementary Fig. 2A and B) and BDNF (ANOVA: *df* _(between groups)_ = 3, *df* _(within groups)_ = 8, *F* = 10.437, *P* = 0.004; *post hoc* test: *P* = 0.012; as shown in Supplementary Fig. 4A and C) were downregulated in the CUS group, and the use of S-ketamine increased the SIRT1 level (*t* test: *t* = 8.623, *P* = 0.001; as shown in Supplementary Fig. 2A and B) and BDNF (*t* test: *t* = 4.855, *P* = 0.008; as shown in Supplementary Fig. 2A and C) protein.


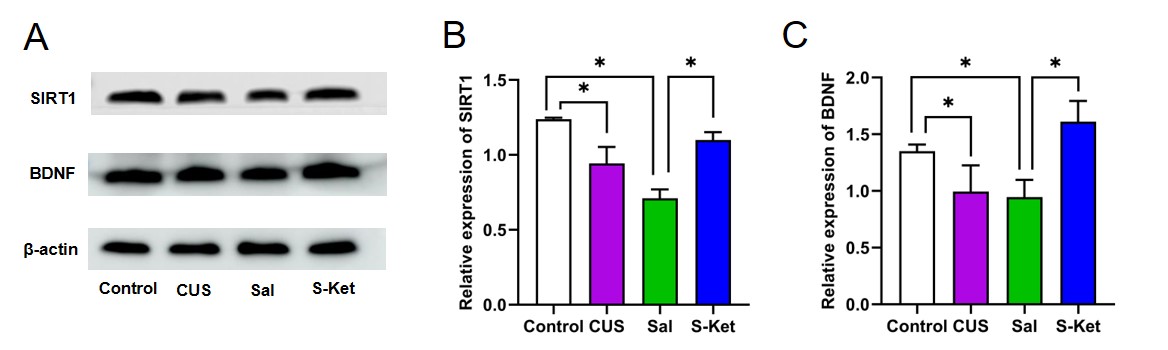


Supplementary Figure 2. S-ketamine upregulated the SIRT1 and BDNF expression in hippocampus. (A) Bands of SIRT1 and BDNF obtained from the Western blot test. (B) Relative expression of SIRT1 protein (n = 3). (C) Relative expression of SIRT1 protein (n = 3). The data are expressed as the mean ± standard error, and *P* <0.05 indicated statistically significant differences.
